# Handbook of Human Anatomy, General, Special, and Topographical

**Published:** 1847-01

**Authors:** 


ART. XXIII.—*Handbook of Human Anatomy, General, Special, and Topographical.* Translated from the original German of DR. ALFRED VON BEHR, and adapted to the use of the English Student, by JOHN BIRKETT, Fellow of the Royal College of Surgeons of England, and Demonstrator of Anatomy at Guy's Hospital.—*London,* 1846. 8vo, pp. 457.

THIS work, according to the author's preface, is intended to serve either as an introduction to the study of anatomy, or for refreshing the memory, more especially of those preparing for examination. For the latter purpose it appears to be well enough suited, but scarcely for the former, and for this reason: the different departments of general, special, and topographical anatomy being all included in the short compass of the work, the descriptions are necessarily extremely brief, and therefore not very intelligible, except to one who has already gone over the subject. Although a brief, it is not an elementary book. The translator, however, is of a different opinion.

In regard to the translation, it is carefully made. Too much pains, however, appear to have been taken to keep by the author's text, whereas the translator would have better accomplished his purpose by adopting the plan and substance of the work merely, and employing his own expressions. By this means the translator would have corrected some inaccuracies of the original, and have avoided some unintelligibilities almost inseparable from an attempt at too close a translation. That the translator was well able for this is shown by the additions which he has himself made.

